# Supplementary material for: Use of RNA and DNA to Identify Mechanisms of Bacterial Community Homogenization
Source: Front Microbiol. 2019 Sep 11;10:2066. doi: 10.3389/fmicb.2019.02066 (PMC6749020; doi:10.3389/fmicb.2019.02066)
Supplement: Supplementary file 1 [file Data_Sheet_1.docx]

|  | **OM** | **P** | **pH** | **K** | **CEC** | **NO_3_-N** | **S** |
| --- | --- | --- | --- | --- | --- | --- | --- |
| **Forest** | 6.95±11.73 | 26.08±12 | 3.9±0.19 | 28.48±15.27 | 1.99±1.41 | 2.62±0.43 | 11.85±1.93 |
| **Burned** | 3.39±0.82 | 83.08±20.97 | 3.98±0.21 | 64.03±15.83 | 4.78±1.29 | 14.34±3.73 | 21.75±3.91 |
| **Plantation** | 2.45±0.49 | 157.08±24.75 | 4.12±0.18 | 47.25±10.02 | 2.76±0.83 | 2.82±1.03 | 13.48±1.57 |

**Supplementary Table 1:** Average soil chemical conditions (with sd) across forest, burned, and plantation sites. Percent organic matter (OM), weak bray phosphorus (P), soil pH (pH), soil potassium (K), cation exchange capacity (CEC), nitrate-nitrogen (NO_3_-N), sulfur (S).


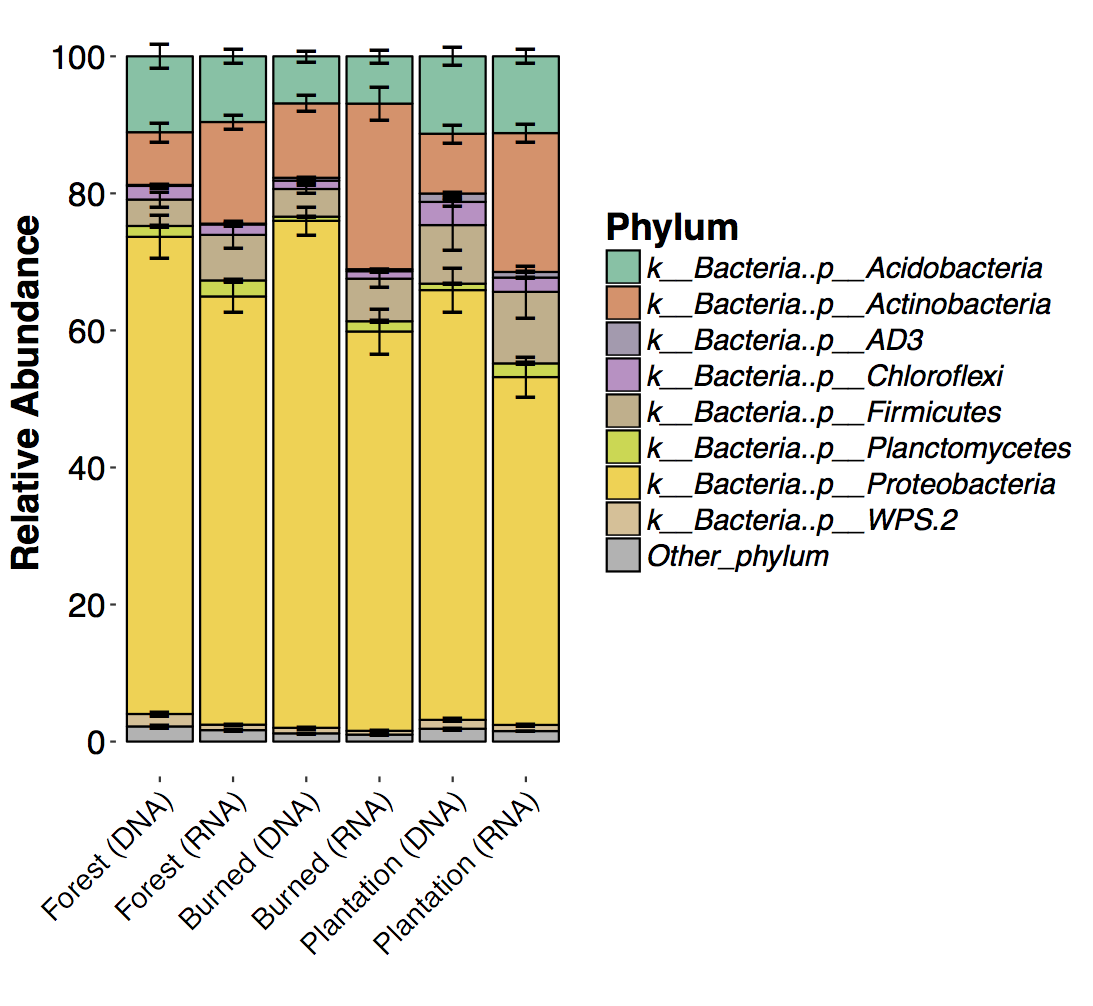


**Supplementary Fig. 1:** Phylum-level composition of bacterial communities inferred by RNA or DNA across forest, burned, and plantation sites.

**
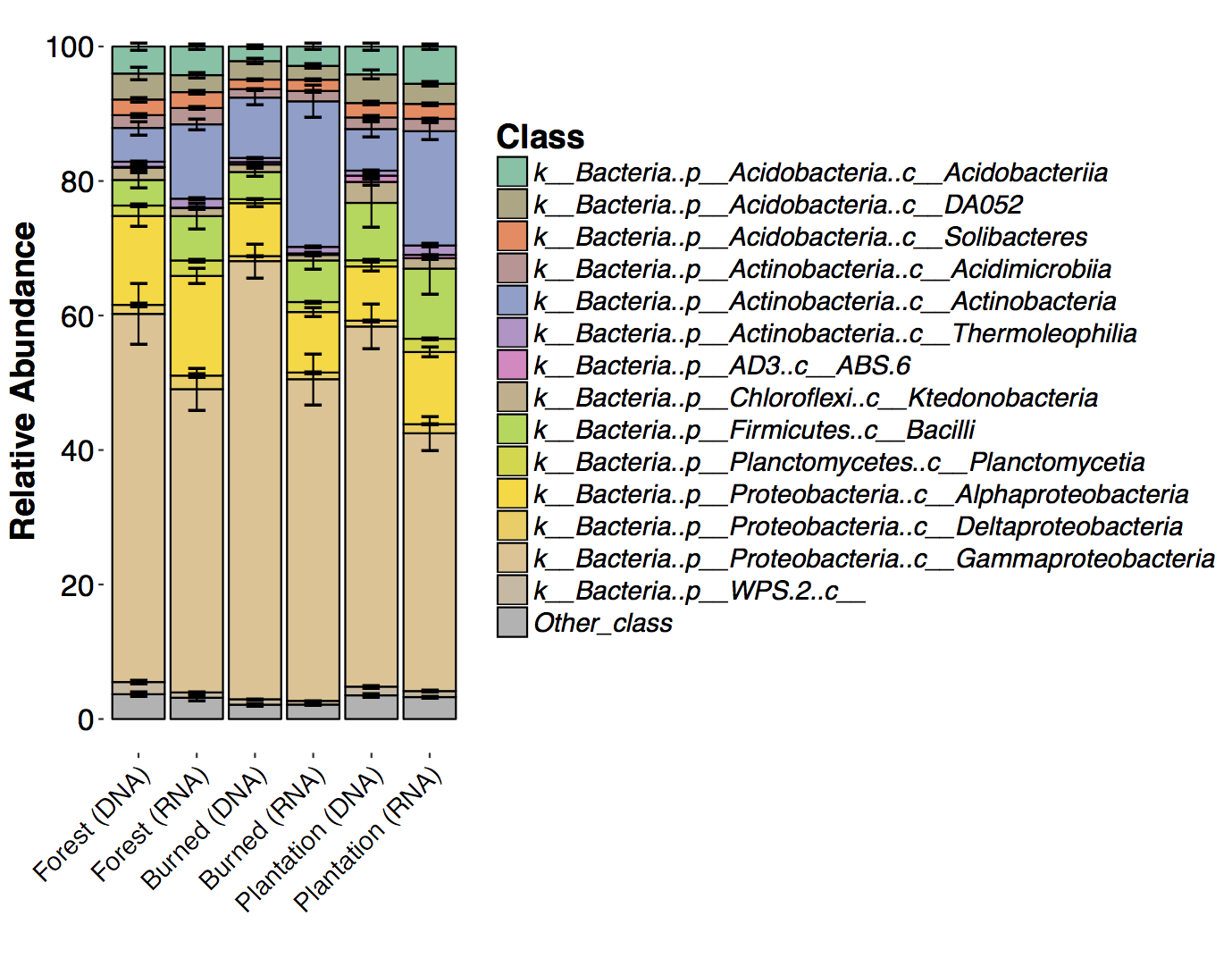
**

**Supplementary Fig. 2:** Class-level composition of bacterial communities inferred by RNA or DNA across forest, burned, and plantation sites.

**
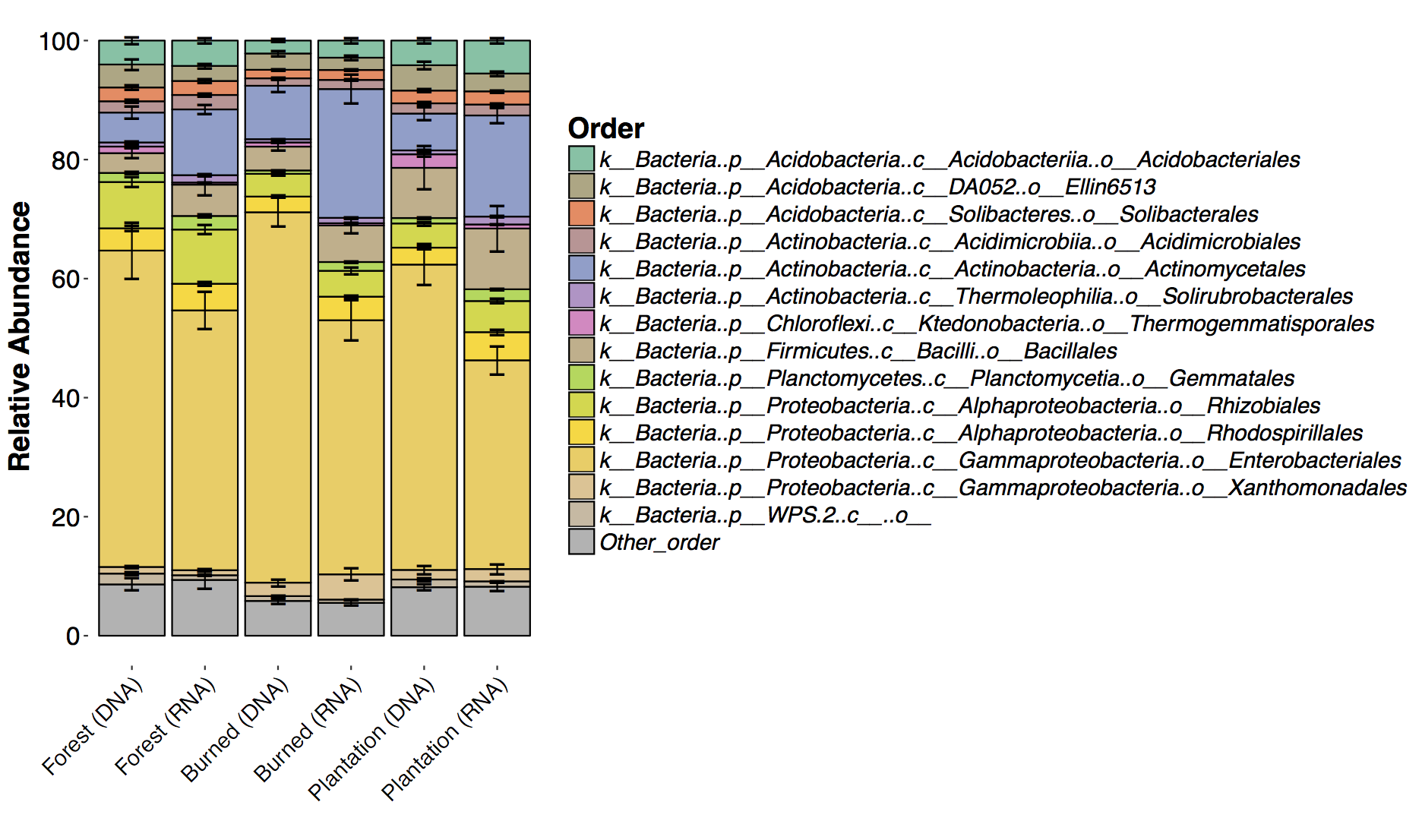
**

**Supplementary Fig. 3:** Order-level composition of bacterial communities inferred by RNA or DNA across forest, burned, and plantation sites.

**
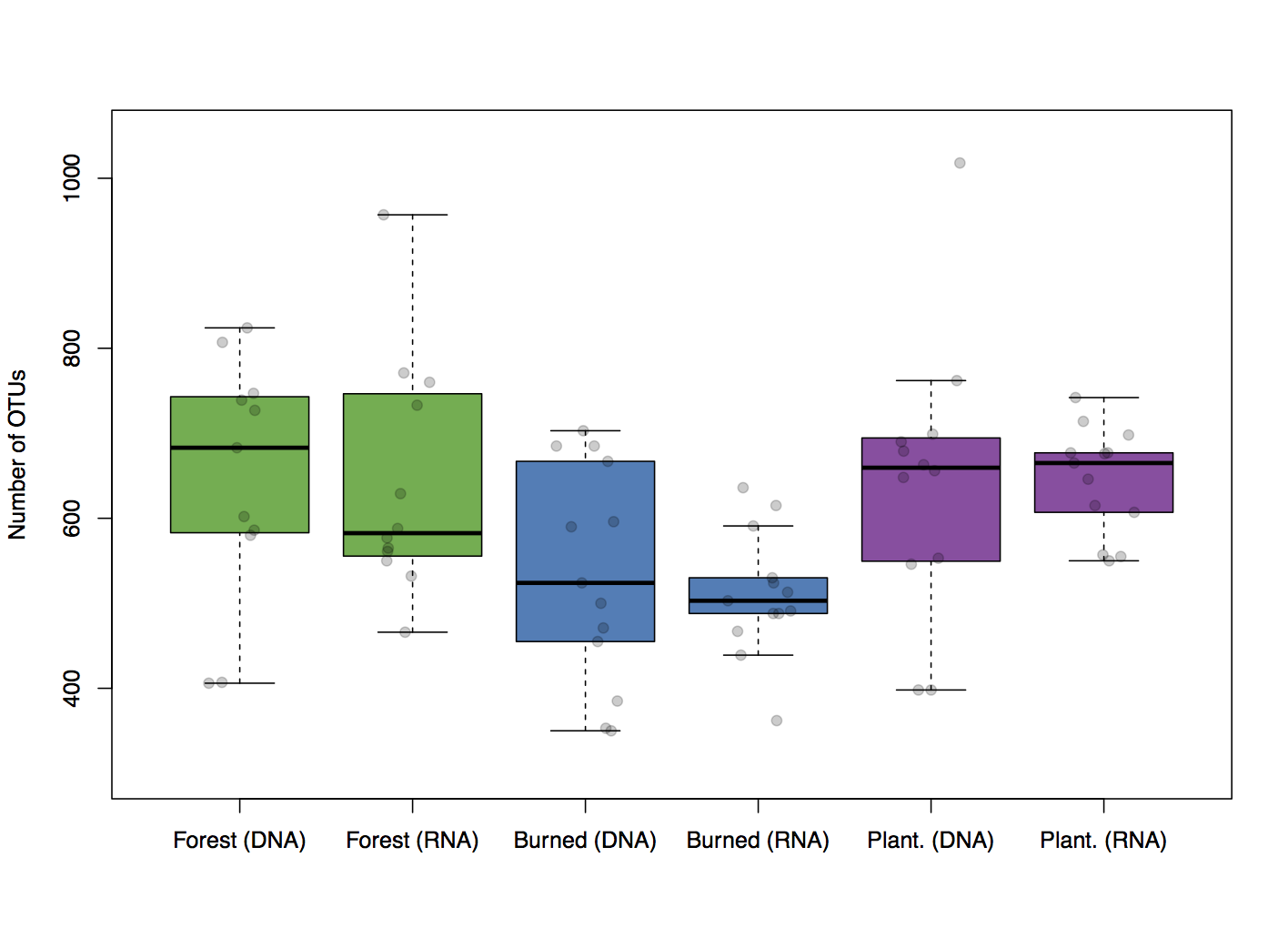
**

**Supplementary Fig. 4:** OTU richness levels of RNA- and DNA-inferred bacterial communities across forest, burned, and plantation sites.

**
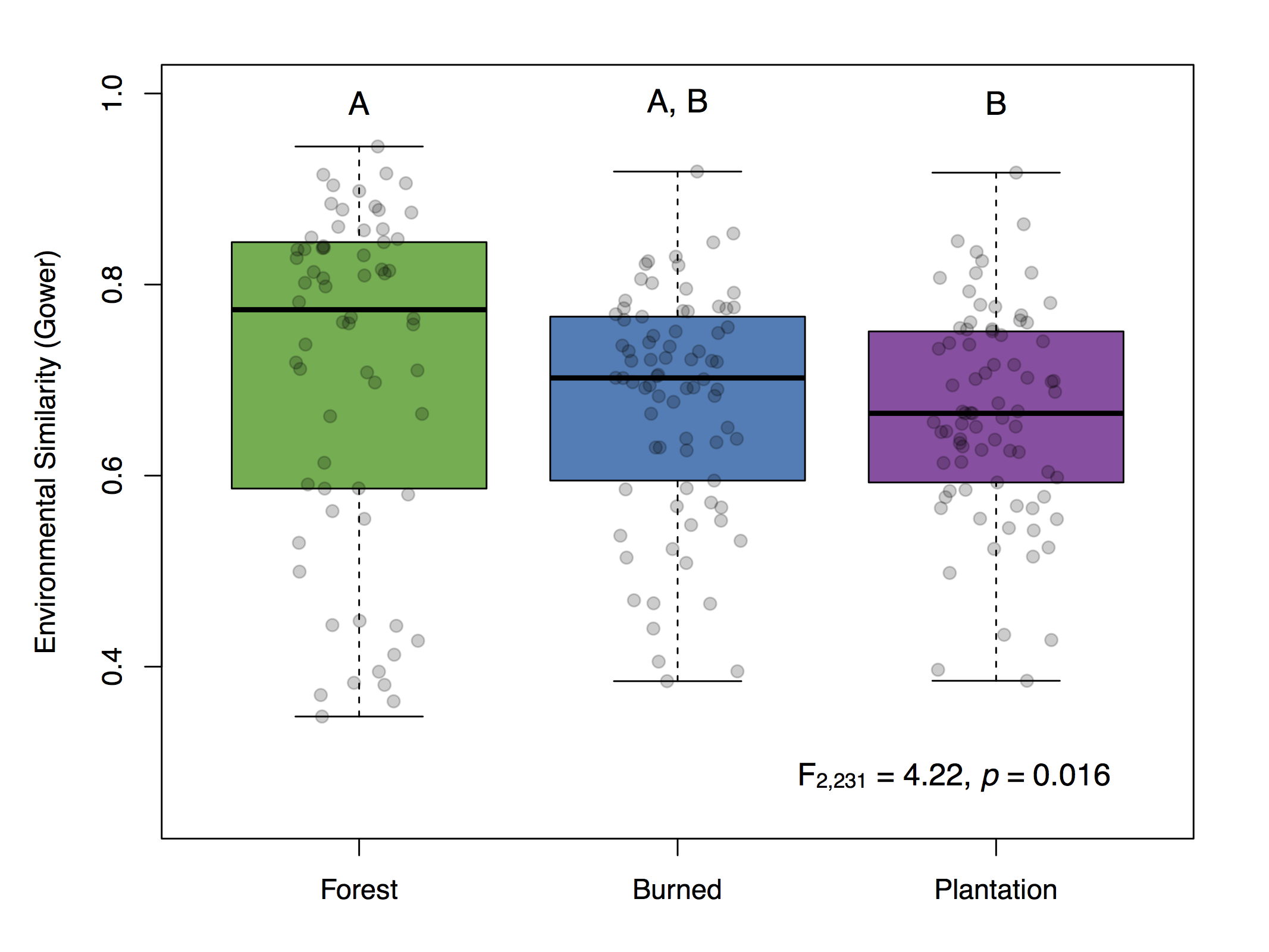
**

**Supplementary Fig. 5:** Average pairwise environmental similarity (1 – Gower) decreases from forest to burned and plantation sites. Different letters correspond to significantly different group means as determined by Tukey’s HSD *p* < 0.05.

**Supplementary Fig. 6:** Average pairwise community similarity (1 – Canberra) levels return to forest levels in burned and plantation sites following the removal of “bloomer” taxa. A) Communities as inferred through DNA, B) communities as inferred through RNA. B. (no bloom) = Burned site with bloomer taxa removed, P. (no bloom) = Plantation site with bloomer taxa removed. Different letters correspond to significantly different group means as determined by Tukey’s HSD *p* < 0.05.
